# Measuring Statistical Dependence via Characteristic Function IPM

**DOI:** 10.3390/e27121254

**Published:** 2025-12-12

**Authors:** Povilas Daniušis, Shubham Juneja, Lukas Kuzma, Virginijus Marcinkevičius

**Affiliations:** 1 Neurotechnology, Laisvės av. 125A, 06118 Vilnius, Lithuania; 2 Research Institute of Natural and Technological Sciences, Vytautas Magnus University, Universiteto Str. 10, Akademija, 53361 Kaunas, Lithuania; 3 Institute of Data Science and Digital Technologies, Vilnius University, Akademijos Str. 4, 08412 Vilnius, Lithuania

**Keywords:** statistical dependence, IPM, characteristic functions, uniform norm, independence testing, supervised feature extraction

## Abstract

We study statistical dependence in the frequency domain using the integral probability metric (IPM) framework. We propose the uniform Fourier dependence measure (UFDM) defined as the uniform norm of the difference between the joint and product-marginal characteristic functions. We provide a theoretical analysis, highlighting key properties, such as invariances, monotonicity in linear dimension reduction, and a concentration bound. For the estimation of the UFDM, we propose a gradient-based algorithm with singular value decomposition (SVD) warm-up and show that this warm-up is essential for stable performance. The empirical estimator of UFDM is differentiable, and it can be integrated into modern machine learning pipelines. In experiments with synthetic and real-world data, we compare UFDM with distance correlation (DCOR), Hilbert–Schmidt independence criterion (HSIC), and matrix-based Rényi’s α-entropy functional (MEF) in permutation-based statistical independence testing and supervised feature extraction. Independence test experiments showed the effectiveness of UFDM at detecting some sparse geometric dependencies in a diverse set of patterns that span different linear and nonlinear interactions, including copulas and geometric structures. In feature extraction experiments across 16 OpenML datasets, we conducted 160 pairwise comparisons: UFDM statistically significantly outperformed other baselines in 20 cases and was outperformed in 13.

## 1. Introduction

The estimation of statistical dependence plays an important role in various statistical and machine learning methods (e.g., hypothesis testing [1], feature selection and extraction [2,3], causal inference [4], self-supervised learning [5], representation learning [6], interpretation of neural models [7], among others). In recent years, various authors (e.g.,  [1,8,9,10,11,12,13,14]) have suggested different approaches to measuring statistical dependence.

In this paper, we focus on the estimation of statistical dependence using characteristic functions (CFs) and integral probability metric (IPM) framework. We propose and investigate a novel IPM-based statistical dependence measure, defined as the uniform norm of the difference between the joint and product-marginal CFs. After introducing core concepts, we conduct a short review of the previous work (Section 2). In Section 3, we formulate the proposed measure and its empirical estimator and perform their theoretical analysis. Section 4 is devoted to empirical investigation. Finally, in Section 5 we discuss results, limitations, and future work. Appendix A contains technical details, such as mathematical proofs, and auxiliary tables. The main contributions of this paper are the following:

- **Theoretical and methodological contributions.** We propose a new IPM-based statistical dependence measure (UFDM) and derive its properties. The main theoretical result of this paper is the structural characterisation of UFDM, which includes invariance under linear transformations and augmentation with independent noise, monotonicity under linear dimension reduction, vanishing under independence, and a concentration bound for its empirical estimator. We additionally propose a gradient-based estimation algorithm with an SVD warm-up to ensure numerical stability.
- **Empirical analysis.** We conduct an empirical study demonstrating the practical effectiveness of UFDM in permutation-based independence testing across diverse linear, nonlinear, and geometrically structured patterns, as well as in supervised feature-extraction tasks on real datasets.

In addition, we provide the accompanying code repository https://github.com/povidanius/UFDM (accessed on 4 December 2025).

### 1.1. IPM Framework

In the context of estimation of statistical dependence, the IPM is a class of metrics between two probability distributions $P_{X,Y}$ and $P_{X}P_{Y}$, defined for a function class F:

(1) $$\mathrm{IPM}\left(P_{X,Y},P_{X}P_{Y}|F\right)=\underset{f\in F}{\sup}\left|E_{U}f\left(U\right)-E_{V}f\left(V\right)\right|,$$

where $U∼P_{X,Y}$, and $V∼P_{X}P_{Y}$ [15].

### 1.2. Characteristic Functions

Let $X\in R^{d_{X}}$, $Y\in R^{d_{Y}}$, and ${\left(X^{T},Y^{T}\right)}^{T}\in R^{d_{X}+d_{Y}}$ be random vectors defined on a common probability space (Ω,F,P). Let us recall that their characteristic functions are given by

(2) $$\phi \left(\alpha \right):=E_{X}e^{i\alpha^{T}X},\;\phi \left(\beta \right):=E_{Y}e^{i\beta^{T}Y},\;\mathrm{and}\;\phi \left(\alpha ,\beta \right):=E_{X,Y}e^{i(\alpha^{T}X+\beta^{T}Y)},$$

where $i^{2}=-1$, $\alpha \in R^{d_{X}}$, and $\beta \in R^{d_{Y}}$. Having *n* i.i.d. realisations ${\left(x_{i},y_{i}\right)}_{i=1}^{n}$, the corresponding empirical characteristic functions (ECFs) are given by

(3) $$\phi_{n}\left(\alpha \right):=\frac{1}{n}\sum_{j=1}^{n}e^{i\alpha^{T}x_{j}},\;\phi_{n}\left(\beta \right):=\frac{1}{n}\sum_{j=1}^{n}e^{i\beta^{T}y_{j}},\;\mathrm{and}\;\phi_{n}\left(\alpha ,\beta \right):=\frac{1}{n}\sum_{j=1}^{n}e^{i(\alpha^{T}x_{j}+\beta^{T}y_{j})}.$$

The uniqueness theorem states that *X* and *Y* have the same distribution if and only if their CFs are identical [16]. Therefore, CFs can be considered a description of a distribution. Alternatively, a CF ϕ can be represented as a real vector $\left(ℜ\phi ,\;ℑ\phi \right)\in R^{2}$, where ℜ and ℑ denote real and imaginary components [17]. This viewpoint avoids explicit reliance on the imaginary unit *i* and makes the geometric structure of CFs more transparent.

For convenience, let us define $\gamma ={\left(\alpha^{T},\beta^{T}\right)}^{T}$, ψ(γ)=ϕ(α)ϕ(β) and let $\psi_{n}\left(\gamma \right)=\psi_{n}\left(\alpha ,\beta \right)$ be its empirical counterpart. In our study, we will utilise IPM framework for investigation of the statistical dependence via

(4) Δ(γ)=ϕ(γ)−ψ(γ)

and its empirical counterpart

(5) $$\Delta_{n}\left(\gamma \right)=\phi_{n}\left(\gamma \right)-\psi_{n}\left(\gamma \right).$$

## 2. Previous Work

Various theoretical instruments have been employed for statistical dependence estimation. For example, weighted $L^{2}$ spaces and CFs (e.g., distance correlation, [13]), reproducing kernel Hilbert spaces (RKHS) (HSIC [1], DIME [18]), information theory (mutual information [19], and generalisations such as MEF [20,21]) and copula theory ([10,22]), among others. Since our work is rooted in the CF-based line of research and IPM framework, and it is empirically evaluated for independence testing and representation learning, let us consider DCOR, HSIC, and MEF, because these three measures form the compact set of high-performing baselines that span CFs, IPMs, and information-theoretic methods, which are widely used in representation learning tasks.

**Distance correlation.** DCOR [13] is defined as

$$\mathrm{DCOR}\left(X,Y\right)=\frac{\mathrm{DCOV}(X,Y)}{\sqrt{\mathrm{DCOV}(X,X)\;\mathrm{DCOV}(Y,Y)}},$$

where the distance covariance (DCOV) is given by

(6) $${\mathrm{DCOV}}^{2}\left(X,Y\right)=\int_{R^{d_{X}+d_{Y}}}{\left|\Delta \left(\gamma \right)\right|}^{2}w\left(\gamma \right)d\gamma ,$$

with weighting function $w\left(\gamma \right)=w\left(\alpha ,\beta \right)=(c_{d_{X}}c_{d_{Y}}{\left||\alpha |\right|}^{1+d_{X}}{\left||\beta |\right|}^{1+d_{Y}}{)}^{-1}$, where $c_{d_{X}}=\pi^{(1+d_{X})/2}/\Gamma \left(\left(1+d_{X}\right)/2\right)$, and $c_{d_{Y}}=\pi^{(1+d_{Y})/2}/\Gamma \left(\left(1+d_{Y}\right)/2\right)$, and Γ(.) is the gamma function. This weighting function allows one to avoid the direct estimation of the integral, expressing it in terms of the covariance of distances between data points [13]. The later result of [23] generalises the distance correlation to multiple random vectors. Given the i.i.d. sample pairs $\left(x_{i},y_{i}\right)$, i=1,…,n, the empirical unbiased estimator of the squared distance covariances [24] is defined as

(7) $${\mathrm{DCOV}}_{n}^{2}\left(X,Y\right)=\frac{1}{n(n-3)}\sum_{i\neq j}A_{ij}B_{ij},$$

where matrices $A=(A_{ij})$, $B=(B_{ij})$ are given by

$$A_{ij}=a_{ij}-\frac{1}{n-2}\sum_{k=1}^{n}a_{ik}-\frac{1}{n-2}\sum_{k=1}^{n}a_{kj}+\frac{1}{\left(n-1)(n-2\right)}\sum_{k,\ell =1}^{n}a_{k\ell },$$

with Euclidean distance $a_{ij}=∥x_{i}-x_{j}∥$. The matrix *B* is defined analogously using distances $b_{ij}=∥y_{i}-y_{j}∥$. The empirical DCOR is then obtained as follows:

$${\mathrm{DCOR}}_{n}\left(X,Y\right)=\frac{{\mathrm{DCOV}}_{n}\left(X,Y\right)}{\sqrt{{\mathrm{DCOV}}_{n}\left(X,X\right)\;{\mathrm{DCOV}}_{n}\left(Y,Y\right)}}.$$

Note that the biased version of the empirical distance-based estimator Equation (7) is equivalent to the ECF-based estimator of Equation (6) (Theorem 1, [13]). While consistency is established for the biased estimator under the moment condition E(∥X∥+∥Y∥)<∞ (Theorem 2, [13]), the unbiased estimator Equation (7) differs only by a finite-sample correction and converges to the same population quantity Equation (6)  [24], implying consistency under the same moment condition.

**HSIC**. For reproducing kernel Hilbert spaces (RKHS) F and G with kernels *k* and *l*, it is defined as

$$\mathrm{HSIC}\left(X,Y\right)=∥E_{XY}k\left(X,\cdot \right)⊗l\left(Y,\cdot \right)-E_{X}k\left(X,\cdot \right)⊗E_{Y}{l\left(Y,\cdot \right)∥}_{\mathrm{HS}}^{2},$$

where ${∥\cdot ∥}_{\mathrm{HS}}$ denotes the Hilbert–Schmidt norm, and ⊗ is the tensor product [1]. Taking a product kernel $\kappa \left(\left(x,y\right),\left(x^{\prime },y^{\prime }\right)\right)=k\left(x,x^{\prime }\right)l\left(y,y^{\prime }\right)$, HSIC is equal to the squared maximum mean discrepancy, which is an instance of an IPM with function class $F=\{f:||f|{|}_{H_{\kappa }}\leq 1\}$, where $H_{\kappa }$ is RKHS generated by κ [25]. Having a sample of paired *n* i.i.d. observations, the empirical estimator is

$${\mathrm{HSIC}}_{n}\left(X,Y\right)=\frac{1}{{\left(n-1\right)}^{2}}\mathrm{tr}\left(KHLH\right)$$

with kernel matrices $K_{ij}=k\left(x_{i},x_{j}\right)$, $L_{ij}=l\left(y_{i},y_{j}\right)$, and centering matrix $H=I-\frac{1}{n}11^{T}$. When both kernels *k* and *l* are translation-invariant (i.e., $k\left(x,x^{\prime }\right)=k_{0}\left(x-x^{\prime }\right)$ on $R^{d_{X}}$ and $l\left(y,y^{\prime }\right)=l_{0}\left(y-y^{\prime }\right)$ on $R^{d_{Y}}$, with $k_{0},l_{0}$ positive definite functions such as the Gaussian $k_{0}\left(v\right)={\exp(-∥v∥}^{2}/\left(2\sigma^{2}\right))$ with σ>0), the product kernel $\kappa \left(\left(x,y\right),\left(x^{\prime },y^{\prime }\right)\right)=k\left(x,x^{\prime }\right)l\left(y,y^{\prime }\right)=k_{0}\left(x-x^{\prime }\right)l_{0}\left(y-y^{\prime }\right)$ is also translation-invariant on $R^{d_{X}+d_{Y}}$. In this case, $\kappa \left(u,v\right)=\kappa_{0}\left(u-v\right)$ for some positive definite function $\kappa_{0}$ on $R^{d_{X}+d_{Y}}$, and HSIC can be expressed in the frequency domain as

(8) $$\mathrm{HSIC}\left(X,Y\right)=\int_{R^{d_{X}+d_{Y}}}{\left|\Delta \left(\gamma \right)\right|}^{2}\;F^{-1}\kappa_{0}\left(\gamma \right)\;d\gamma ,$$

where $\gamma ={\left(\alpha^{T},\beta^{T}\right)}^{T}$, and $F^{-1}\kappa_{0}$ denotes the inverse Fourier transform of $\kappa_{0}$. Therefore, for translation-invariant kernels, HSIC is structurally analogous to distance covariance, since it also corresponds to the squared $L^{2}$ norm of Δ (Equation (4)), with weighting determined by κ.

**MEF.** Shannon mutual information is defined by $\mathrm{MI}\left(X,Y\right)=E_{X,Y}\log\frac{p(X,Y)}{p(X)p(Y)}$ [19]. The neural estimation of mutual information (MINE, [26]) uses its variational (Donsker–Varadhan) representation $\mathrm{MI}\left(X,Y\right)\approx max_{\theta }E_{X,Y}f\left(x,y|\theta \right)-\log\left(E_{X}E_{Y}e^{f(x,y|\theta )}\right)$, since it allows avoiding density estimation (here f(x,y|θ) is a neural network with parameters θ). In this case, the optimisation is performed over the space of neural network parameters, which often leads to unstable training and biased estimates due to the unboundedness of the objective and the difficulty of balancing the exponential term. The matrix-based Rényi’s α-order entropy functional (MEF) [20,21,27] provides a kernel version of mutual information that avoids both density estimation and neural optimization. For random variables *X* and *Y* with distributions $P_{X}$, $P_{Y}$, and $P_{XY}$, it is defined as

(9) $${\mathrm{MEF}}_{\alpha }\left(X,Y\right)=S_{\alpha }\left(P_{X}\right)+S_{\alpha }\left(P_{Y}\right)-S_{\alpha }\left(P_{XY}\right),$$

where $S_{\alpha }\left(P_{X}\right)=\frac{1}{1-\alpha }\log_{2}\left(\mathrm{tr}\left(T_{X}^{\alpha }\right)\right)$ and $T_{X}$ is the normalised kernel integral operator on $L^{2}\left(P_{X}\right)$ [27]. Given i.i.d. samples ${\left\{\left(x_{i},y_{i}\right)\right\}}_{i=1}^{n}$ with Gram matrices $K_{ij}=k\left(x_{i},x_{j}\right)$ and $L_{ij}=l\left(y_{i},y_{j}\right)$, the empirical estimator is

(10) $${\mathrm{MEF}}_{\alpha ,n}\left(X,Y\right)=S_{\alpha ,n}\;\left(\frac{K}{\mathrm{tr}(K)}\right)+S_{\alpha ,n}\;\left(\frac{L}{\mathrm{tr}(L)}\right)-S_{\alpha ,n}\;\left(\frac{K⊙L}{\mathrm{tr}(K⊙L)}\right),$$

where ⊙ denotes the element-wise product, $S_{\alpha ,n}\left(A\right)=\frac{1}{1-\alpha }\log_{2}\;\left(\sum_{i}\lambda_{i}{\left(A\right)}^{\alpha }\right)$, and $\lambda_{i}$ are eigenvalues of n×n matrix *A*.

### Motivation

The motivation of our work stems from the theoretical observation that applying the $L^{\infty }$ norm to Δ Equation (4) yields a novel, structurally simple IPM with some advantageous properties, such as the ability to detect arbitrary statistical dependencies, invariance under full-rank linear transformations and coordinate augmentation with independent noise, and monotonicity under linear dimension reduction (Theorem 1).

Since the $L^{\infty }$ norm isolates the most informative frequencies where dependence concentrates, we hypothesise that its empirical estimator could extract important structure from Δ that may be diluted by weighted $L^{2}$ or other global approaches such as DCOV, HSIC, and MEF.

## 3. Proposed Measure

Given two random vectors *X* and *Y* of dimensions $d_{X}$ and $d_{Y}$, and assuming possibly unknown joint distribution $P_{X,Y}$, we define our measure via IPM with function class $F=\{f:f\left(z\right)=e^{i\gamma^{T}z};\gamma ,z\in R^{d_{X}+d_{Y}},i^{2}=-1\}$, which corresponds to the following.

**Definition** **1.** *Uniform Fourier Dependence Measure.*

(11) $$\mathrm{UFDM}\left(X,Y\right)={\left||\Delta |\right|}_{L_{\infty }}=\underset{\gamma }{\sup}\left|\Delta \left(\gamma \right)\right|.$$

Since CF is a Fourier transform of a probability distribution, and the norm in $L^{\infty }$ is called a uniform norm, we refer to it as Uniform Fourier Dependence Measure (UFDM).

**Theorem** **1.** *UFDM has the following properties:*

1. *0≤UFDM(X,Y)≤1.*
2. *UFDM(X,Y)=UFDM(Y,X).*
3. UFDM(X,Y)=0 *if and only if* X⊥Y *(*⊥ *denotes statistical independence).*
4. *For Gaussian random vectors $X∼N(0,\Sigma_{X})$, $Y∼N(0,\Sigma_{Y})$ with cross-covariance matrix $\Sigma_{X,Y}$ we have $\mathrm{UFDM}\left(X,Y\right)=\sup_{\alpha ,\beta }e^{-\frac{1}{2}\left(\alpha^{T}\Sigma_{X}\alpha +\beta^{T}\Sigma_{Y}\beta \right)}\left|e^{-\alpha^{T}\Sigma_{X,Y}\beta }-1\right|$.*
5. *Invariance under full-rank linear transformation: UFDM(AX+a,BY+b)=UFDM(X,Y) for any full-rank matrices $A\in R^{d_{X}\times d_{X}}$, $B\in R^{d_{Y}\times d_{Y}}$ and vectors $a\in R^{d_{X}}$, $b\in R^{d_{Y}}$.*
6. *Linear dimension reduction does not increase UFDM(X,Y).*
7. *If X⊥E, for any continuous function $f:R^{d_{X}}\to R^{d_{Y}}$, $\lim_{\lambda \to \infty }\mathrm{UFDM}\left(X,f\left(X\right)+\lambda E\right)=0$, if E has a density.*
8. *If X and Y have densities, then $\mathrm{UFDM}\left(X,Y\right)\leq \min\left\{1,\sqrt{2\mathrm{MI}(X,Y)}\right\}$, where MI(X,Y) is mutual information.*
9. *Invariance to augmentation with independent noise: let X,Y,Z be random vectors such that $Z⊥{\left(X^{⊤},Y^{⊤}\right)}^{⊤}$. Then $\mathrm{UFDM}\left({\left(X^{⊤},Z^{⊤}\right)}^{⊤},\;Y\right)=\mathrm{UFDM}\left(X,Y\right)$.*

**Proof.**  See Appendix A.1.    □

**Interpretation of UFDM via canonical correlation analysis (CCA).** In the Gaussian case, the UFDM objective reduces analytically to CCA via a closed-form expression (Theorem 1, Property 4): after whitening (setting $u=\Sigma_{X}^{1/2}\alpha$ and $v=\Sigma_{Y}^{1/2}\beta$), it becomes $\max_{u,v}e^{-\frac{1}{2}{\left(|u\right|}^{2}+{\left|v\right|}^{2})}\left(1-e^{-u^{⊤}Kv}\right)$, where $K=\Sigma_{X}^{-1/2}\Sigma_{XY}\Sigma_{Y}^{-1/2}$. By von Neumann’s inequality, the maximizers (u,v) align with the leading singular vectors of *K*, corresponding to the top CCA pair. Note that since Gaussian independence is equivalent to the vanishing of the leading canonical correlation $\rho_{1}$ (as all remaining correlations $0\leq \rho_{j}\leq \rho_{1}$, j>1 must also vanish), UFDM’s focus on the leading canonical correlation entails no loss of discriminatory power.
**Interpretation of UFDM via cumulants.** Let us recall that $\gamma ={\left(\alpha^{T},\beta^{T}\right)}^{T}$, ϕ(γ)=ϕ(α,β), ψ(γ)=ϕ(α)ϕ(β). For general distributions, writing Δ(γ)=ψ(γ)(exp(C(γ))−1) offers a cumulant-series factorization, with $C\left(\gamma \right)=\log\frac{\phi (\gamma )}{\psi (\gamma )}=\sum_{p,q\geq 1}\frac{i^{p+q}}{p!q!}\langle \kappa_{p,q},\alpha^{⊗p}⊗\beta^{⊗q}\rangle$, where $\kappa_{p,q}$ are cross-cumulants and $\alpha^{⊗p}⊗\beta^{⊗q}$ are the (p+q)-order tensors formed by the tensor product of *p* copies of α and *q* copies of β. The leading term, corresponding to p=q=1, is $\frac{i^{2}}{1!,1!}\langle \kappa_{1,1},\alpha ⊗\beta \rangle =-\alpha^{⊤}\Sigma_{XY}\beta$ (with $\kappa_{1,1}=\Sigma_{XY}$ for centered variables), which aligns with the CCA interpretation, while higher-order $\kappa_{p,q}$ terms capture non-Gaussian deviations, interpreting UFDM as a frequency-domain approach that aligns (α,β) with cross-cumulant directions under marginal damping by ψ(γ).
**Remark on the representations of CFs.** Since $\mathrm{UFDM}\left(X,Y\right)=\sup_{\gamma }{∥\left(ℜ\Delta \left(\gamma \right),\;ℑ\Delta \left(\gamma \right)\right)∥}_{2}$, the UFDM objective naturally operates on the real two-dimensional vector formed by the real and imaginary parts of Δ(γ). This aligns with recent work on real-vector representations of characteristic functions [17] and shows that UFDM does not rely on any special algebraic role of the imaginary unit.

### 3.1. Estimation

Having i.i.d. observations $\left(X^{n},Y^{n}\right)=\left(x_{j},y_{j}\right)∼P_{X,Y}$, j=1,2,…,n, we define and discuss empirical estimators of UFDM. Recall that (Section 1.2) that $\gamma ={\left(\alpha^{T},\beta^{T}\right)}^{T}$ and let ϕ(α),ϕ(β), and ϕ(γ) be CFs of *X*, *Y*, and (X,Y), respectively ($\alpha \in R^{d_{X}}$, $\beta \in R^{d_{Y}}$, and $\gamma \in R^{d_{X}+d_{Y}}$). Let us also denote norms ${\left||f|\right|}_{L_{\infty }}^{t}=\sup_{||\tau ||<t}\left|f\left(\tau \right)\right|$, ${\left||f|\right|}_{L_{\infty }}=\sup_{\tau }\left|f\left(\tau \right)\right|$, for t>0 and multivariate τ.

**Empirical estimator.** Let us define the empirical estimator of UFDM for a fixed t>0:

(12) $${\mathrm{UFDM}}_{n}^{t}\left(X^{n},Y^{n}\right)=\left|\right|\Delta_{n}{\left|\right|}_{L_{\infty }}^{t}.$$

### 3.2. Estimator Convergence

The ECF is a uniformly consistent estimator of CF in each bounded subset [28] (i.e., $\lim_{n\to \infty }\sup_{||\gamma ||<t}\left|\phi \left(\gamma \right)-\phi_{n}\left(\gamma \right)\right|=0$ almost surely for any fixed t>0) [28]. By the triangle inequality, this implies the following:

**Proposition** **1.** *For a fixed t>0, $\lim_{n\to \infty }\left|\right|\Delta_{n}-\Delta {\left|\right|}_{L_{\infty }}^{t}=0$, almost surely.*

**Theorem** **2** ([29])**.** *If $t_{n}\to \infty$ and $\frac{\log t_{n}}{n}\to 0$, as n→∞, then $\lim_{n\to \infty }\sup_{||\gamma ||<t_{n}}\left|\xi \left(\gamma \right)-\xi_{n}\left(\gamma \right)\right|=0$ almost surely for any CFξ(γ) and corresponding ECF$\xi_{n}\left(\gamma \right)$.*

This implies the convergence of the empirical estimator Equation (12):

**Proposition** **2.** *If $t_{n}\to \infty$ and $\frac{\log t_{n}}{n}\to 0$, as n→∞, then $\lim_{n\to \infty }\left|\right|\Delta_{n}{\left|\right|}_{L_{\infty }}^{t_{n}}=\mathrm{UFDM}\left(X,Y\right)$, almost surely.*

**Proof.**  See Appendix A.1.    □

Note that ECF does not converge to CF [28,29] uniformly in the entire space. Therefore, to ensure the convergence of the empirical estimator of UFDM, we need to bound the norm by slowly growing balls as in Theorem 2. The finite–sample analysis of the convergence of empirical UFDM Equation (12) to its truncated population counterpart (${\mathrm{UFDM}}^{t}\left(X,Y\right)={\left||\Delta |\right|}_{L_{\infty }}^{t}$) yields the following concentration inequality.

**Theorem** **3.** *Let us assume that ${E||X||}^{2}<\infty$, ${E||Y||}^{2}<\infty$. Let us define $d=d_{X}+d_{Y}$, $Z={\left(X^{T},Y^{T}\right)}^{T}$, and W=||X||+||Y||+||Z||. Then there exists a constant C, such that for every fixed $\varepsilon >\frac{1}{n}$, t>0:*

$$\mathrm{Pr}\;\left(\;|{\mathrm{UFDM}}_{n}^{t}\left(X^{n},Y^{n}\right)-{\mathrm{UFDM}}^{t}\left(X,Y\right)|>\varepsilon \right)\;\leq \;2{\left(\frac{Ct}{\varepsilon }\right)}^{d}\exp\;\left(-\;\frac{n}{18}\;{\left(\frac{\varepsilon }{2}-\frac{1}{n}\right)}^{\;2}\right)+\frac{\sigma^{2}}{nL^{2}},$$

*where L=EW, and $\sigma^{2}=E{\left(W-L\right)}^{2}$.*

**Proof.**  See Appendix A.2.    □

### 3.3. Estimator Computation

In practice, UFDM can be estimated iteratively using Algorithm 1. Since it depends on initial parameters α and β, the complementary Algorithm 2 is designed for their data-driven initialisation. According to our experience with UFDM applications, Algorithm 2 is very important, since without it we often encountered stability issues, and initially had to rely on various heuristics, such as parameter normalisation to the unit sphere. In our opinion, this is because $\Delta_{n}$ is a highly nonlinear optimisation surface (especially in larger dimensions), which complicates the finding of the corresponding maxima.
**Algorithm 1** UFDM estimation**Require:** Number of iterations *N*, batch size $n_{b}$, initial $\alpha \in R^{d_{X}},\beta \in R^{d_{Y}}$.    **for** iteration=1 to *N* **do**          Sample batch $\left(X^{n_{b}},Y^{n_{b}}\right)={\left(x_{i},y_{i}\right)}_{i=1}^{n_{b}}$.          Standardise $\left(X^{n_{b}},Y^{n_{b}}\right)$ to zero mean and unit variance.          $\alpha ,\beta \leftarrow \mathrm{AdamW}(\left[\alpha ,\beta \right],-|\Delta_{n_{b}}\left(\alpha ,\beta \right)|)$.    **end for**    **return** Δ(α,β), α, β

**Algorithm 2** SVD warm-up**Require:**
Batch size $n_{b}$.    Sample batch $\left(X^{n_{b}},Y^{n_{b}}\right)={\left(x_{i},y_{i}\right)}_{i=1}^{n_{b}}$.    Compute cross-covariance $C={\left(X^{n_{b}}\right)}^{⊤}Y^{n_{b}}/n_{b}$.    Decompose: $\left[U,\Sigma ,V^{H}\right]=\mathrm{SVD}\left(C\right)$.    $\alpha \leftarrow U_{:,1},\;\beta \leftarrow V_{1,:}^{H,⊤}$.    **return** α, β

The computational complexity of Algorithm 2 consists of cross-covariance computation and finding its SVD a complexity of $O\left(n_{b}d_{X}d_{Y}+d_{X}d_{Y}\min\left(d_{X},d_{Y}\right)\right)$. Having initialisation of α and β, the complexity of Algorithm 1 is $O\left(N\;n_{b}\;\left(d_{X}+d_{Y}\right)\right)$. Hence, the total computational complexity of the sequential application of Algorithm 2 and Algorithm 1 is $O\left(n_{b}d_{X}d_{Y}+d_{X}d_{Y}\min\left(d_{X},d_{Y}\right)+N\;n_{b}\;\left(d_{X}+d_{Y}\right)\right)$. Finally, having the optimal $\alpha^{*}$ and $\beta^{*}$ computed by Algorithm 1, the evaluation of empirical UFDM has computational complexity linear in sample size.

## 4. Experiments

For UFDM, we used SVD warm-up (Algorithm 2) for parameter initialisation and fixed truncation parameter *t* to 25.0. For kernel measures, HSIC and MEF, we used Gaussian kernels for both *X* and *Y*, with a bandwidth selected using median heuristics [30]. For MEF measure α was set to 1.01, as in [21].

### 4.1. Permutation Tests

**Permutation tests with UFDM.** We compared UFDM, DCOR, HSIC, and MEF in permutation-based statistical independence testing ($H_{0}:X⊥Y$ versus the alternative H1:X⊥Y) using a set of multivariate distributions. We investigated scenarios with a sample size of n=750 and data dimensions d∈{5,15,25} ($d_{X}=d_{Y}=d$). To ensure valid finite-sample calibration, permutation *p*-values were computed with the Phipson–Smyth correction [31].
**Hyperparameters.** We used 500 permutations per *p*-value. The number of iterations in UFDM estimation Algorithm 1 was set to 100. The batch size equaled the sample size (n=750). We used a learning rate of 0.025. Due to the high computation time (permutation tests took ≈6.3 days on five machines with Intel i7 CPU, 16GB of RAM, and Nvidia GeForce RTX 2060 12 GB GPU), we relied on 500 *p*-values for each test in the $H_{0}$ scenario and on 100 *p*-values for each test in the $H_{1}$ scenario.
**Distributions analysed.** In the $H_{0}$ case, *X* was sampled from multivariate uniform, Gaussian, and Student t(3) distributions (corresponding to no-tail, light-tail, and heavy-tail scenarios, respectively), and *Y* was independently sampled from the same set of distributions. Afterwards, we examined the uniformity of the *p*-values obtained from permutation tests using different statistical measures, through QQ-plots and Kolmogorov–Smirnov (KS) tests.

In the $H_{1}$ case, *X* and *Y* were related through statistical dependencies described in Table A2. These dependencies include structured dependence patterns, where *X* was sampled from the same set of distributions (multivariate uniform, Gaussian, and Student t(3)), and *Y* was generated as Y=f(X)+0.1ϵ, with ϵ denoting additive Gaussian noise independent of *X*. We also examined more complex dependencies (Table A2), where the relationship between *X* and *Y* was modeled using copulas, bimodal, circular, and other nonlinear patterns. Using this setup, we evaluated the empirical power of the permutation tests based on the same collection of statistical measures.

**Figure 1:**
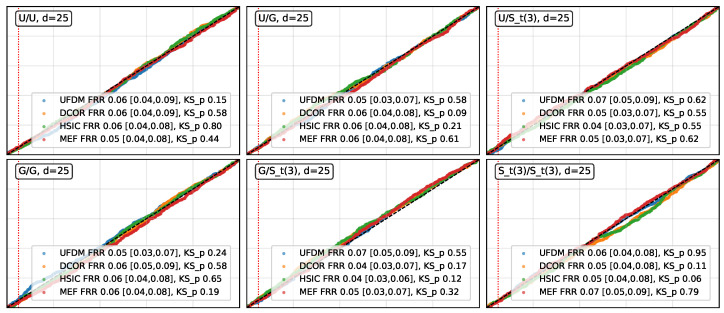

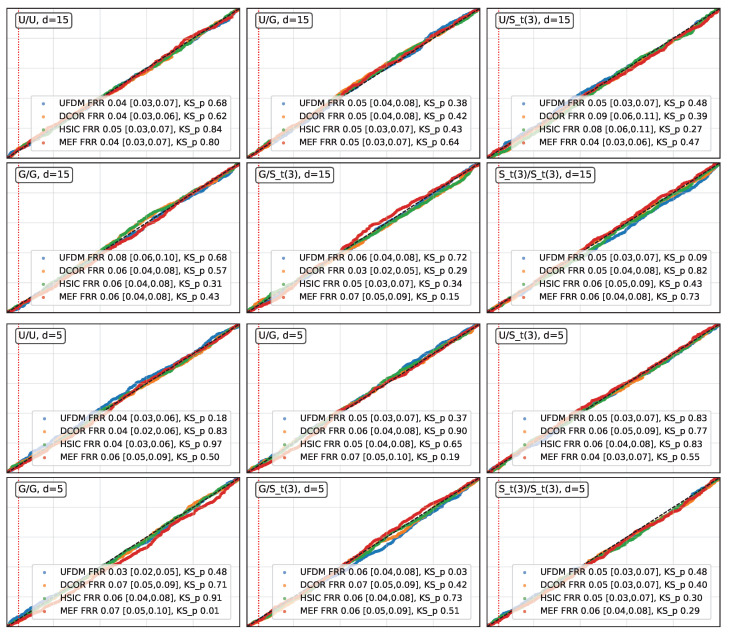
Empirical QQ-plots of *p*-values under $H_{0}$. The dashed vertical line corresponds to the nominal significance level 0.05. The empirical FRR and its Wilson confidence interval, *p*-values of KS test are reported in the legend.

**Results for $H_{0}$.** As shown in Figure 1, UFDM, DCOR, HSIC, and MEF exhibited approximately uniform permutation *p*-values across all distribution pairs and dimensions, with empirical false rejection rates (FRR) remaining close to the nominal 0.05 level. Isolated low KS *p*-values below 0.05 occurred in only two cases: one for MEF in the Gaussian/Gaussian pair at dimension 5 (*p*-value of 0.01) and one for UFDM in the Gaussian/Student-t pair at dimension 5 (*p*-value of 0.03), suggesting minor sampling variability rather than systematic deviations from uniformity. These results show that UFDM remained comparably stable to DCOR, HSIC and MEF, in terms of type-I error control under $H_{0}$.
**Results for $H_{1}$.** The empirical power and its 0.95-Wilson confidence intervals (CIs) are presented in Table 1 and Table 2. These results show that, in most cases, the empirical power of UFDM, DCOR, HSIC, and MEF was approximately equal to 1.00. However, Table 2 also reveals that for the sparse *Circular* and *Interleaved Moons* patterns (d≥15), MEF exhibited a noticeable decrease in empirical power. We conjecture that this reduction may stem from MEF’s comparatively higher sensitivity to kernel bandwidth selection in these specific, geometrically structured patterns. On the other hand, UFDM’s robustness in these settings may also be explained by its *invariance to augmentation with independent noise* (Theorem 1, Property 9), which helps to preserve the detectability of sparse geometric dependencies embedded within high-dimensional noise coordinates.
**Ablation experiment.** The necessity of the SVD warm-up (Algorithm 2) is empirically demonstrated in Table A1, where the *p*-values obtained without SVD warm-up systematically fail to reveal dependence in many nonlinear patterns.
**Remark on the stability of the estimator.** Since the UFDM objective is non-convex, different random initialisations may potentially lead to distinct local optima. To assess the impact of this issue, we investigated the numerical stability of the UFDM estimator. We computed the mean and standard deviation of the statistic across 50 independent runs for each distribution pattern and dimension (Table 1 and Table 2), as well as for the corresponding permuted patterns in which dependence is destroyed, as reported in Table 3. The obtained results align with the permutation test findings. While a slight upward shift is observed under independent (permuted) data, the proposed estimator retained consistent separation between dependent and independent settings and exhibited stable behaviour across random restarts.

### 4.2. Supervised Feature Extraction

Feature construction is often a key initial step in machine learning with tabular data. These methods can be roughly classified into feature selection and feature extraction. Feature selection identifies a subset of relevant inputs, either incrementally (e.g., via univariate filters) or through other strategies, and feature extraction transforms inputs into lower-dimensional, informative representations. In our experiments, we used the latter approach because of its computational effectiveness. The total computational time for these experiments was ≈94.3 h on single Intel i7 CPU, 16GB of RAM, and Nvidia GeForce RTX 2060 12 GB GPU machine.

Let ${\left(x_{i},y_{i}\right)}_{i=1}^{n}$ be a classification dataset consisting of *n* pairs of $d_{X}$-dimensional inputs $x_{i}$, and $d_{Y}$-dimensional one-hot encoded outputs $y_{i}$. In our experiments, we used a collection of OpenML classification datasets [32], which cover different domains, input and output dimensionalities. We randomly split the data into training, validation, and test sets using the proportions (0.5,0.1,0.4), respectively. We followed the dependence maximisation scheme (e.g., [3,33]) by seeking

(13) $$W^{*}=arg\max_{W}\mathrm{DEP}\left(Wx,y\right)-\lambda \mathrm{tr}\left({\left(W^{T}W-I\right)}^{T}\left(W^{T}W-I\right)\right),\;$$

where DEP∈{UFDM,DCOR,HSIC,MEF}. To evaluate the obtained features $f\left(x\right)=W^{*}x$, we used logistic regression’s [34] accuracy, measured on the test set. For each baseline method, we selected the dimensions of the features that correspond to the maximal validation accuracy of the investigated method, checking all dimensions starting from 1 with a step of 10% of $d_{X}$. Similarly, we selected λ∈{0.1,1.0,10.0}. The feature extraction loss Equation (13) was optimised via Algorithm 1 for 100 epochs, with the learning rate set to 0.025, as in permutation testing experiments (Section 4.1).

**Baselines.** We compared the following baselines: unmodified inputs (denoted as RAW); and Equation (13) scheme with dependence measures: UFDM, DCOR, MEF, and HSIC. We also included the neighbourhood component analysis (NCA) [35] baseline, which is specially tailored for classification.
**Evaluation metrics.** Let us denote $a_{r,p}\left(b,b^{\prime }|d\right)=1$, if for *r* runs on the dataset *d* the average test set accuracy of baseline *b* is statistically significantly higher than that of $b^{\prime }$ with *p*-value threshold *p*. For statistical significance assessment, we used Wilcoxon’s signed-rank test [36]. We computed the win ranking (WR) and loss ranking (LR) as

(14) $$\mathrm{WR}\left(b\right)=\sum_{d}\sum_{b^{\prime }\neq b}a_{25,0.05}\left(b,b^{\prime }|d\right)\;\mathrm{and}\;\mathrm{LR}\left(b\right)=\sum_{d}\sum_{b^{\prime }\neq b}a_{25,0.05}\left(b^{\prime },b|d\right).\;$$

Based on these metrics, Table 4 includes full information on how many cases each baseline method statistically significantly outperformed the other method.
**Results.** Using 18 datasets, we conducted 80 feature efficiency evaluations (excluding the RAW baseline) and 160 feature efficiency comparisons, of which 97 (∼60%) were statistically different. The results of the feature extraction experiments are presented in Table 4 and Table 5. They reveal that, although MEF showed best WR, UFDM also performed comparable to other measures: it statistically significantly outperformed them in 6+4+5+5=20 cases (listed in Table 6), and was outperformed in 2+4+2+5=13 cases (Table 4).

In addition to pairwise statistical comparisons using Wilcoxon’s test, we also conducted statistical analysis to clarify whether some method is globally better or worse over multiple datasets using the methodology described in [37]. In this analysis, the Friedman/Iman–Davenport test (α=0.05) showed a global significant difference between the five methods. The Nemenyi post hoc test (α=0.05, critical difference 1.884) revealed that RAW was significantly outperformed by the other methods; however, it also showed the absence of a global best-performing method.

## 5. Conclusions

**Results.** We proposed and analysed an IPM-based statistical dependence measure, UFDM, defined as the $L^{\infty }$ norm of the difference between the joint and product-marginal characteristic functions. UFDM applies to pairs of random vectors of possibly different dimensions and can be integrated into modern machine learning pipelines. In contrast to global measures (e.g., DCOR, HSIC, MEF), which aggregate information across the entire frequency domain, UFDM identifies spectrally localised dependencies by highlighting frequencies where the discrepancy is maximised, thereby offering potentially interpretable insights into the structure of dependence. We theoretically established key properties of UFDM, such as invariance under linear transformations and augmentation with independent noise, monotonicity under dimension reduction, and vanishing under independence. We also showed that UFDM’s objective aligns with the vectorial representation of CFs. In addition, we investigated the consistency of the empirical estimator and derived a finite-sample concentration bound. For practical estimation, we proposed a gradient-based estimation algorithm with SVD warm-up, and this warm-up was found to be essential for stable convergence.

We evaluated UFDM on simulated and real data in permutation-based independence testing and supervised feature extraction. The permutation test experiments (n=750, d∈{5,15,25}) indicated that in this regime UFDM performed comparably to established baseline measures, exhibiting similar empirical power and calibration across diverse dependence structures. Notably, UFDM maintained high power on the *Circular* and *Interleaved Moons* datasets, where some other measures displayed reduced sensitivity under these geometrically structured dependencies. These findings suggest that UFDM provides a complementary addition to the family of widely used dependence measures (DCOR, HSIC, and MEF).

Further experiments with real data demonstrated that, in dependence-based supervised feature extraction, UFDM often performed on par with the well-established alternatives (HSIC, DCOR, MEF) and with NCA, which is specifically designed for classification. Across 16 datasets and 160 pairwise comparisons, UFDM statistically significantly outperformed other baselines in 20 cases and was outperformed in 13. To facilitate reproducibility, we provide an open-source repository.

**Limitations.** Computing UFDM requires maximising a highly nonlinear objective, which makes the estimator sensitive to initialisation and optimisation settings. Although the proposed SVD warm-up substantially improves numerical stability, estimation may still become more challenging as dimensionality *d* increases or sample size *n* decreases. From the perspective of the effective (n,d), our empirical evaluation covers two different tasks. First, in independence testing with synthetic data and n=750 and d∈{5,15,25}, UFDM maintained effectiveness across diverse dependence structures. Our preliminary experiments with n=375,d∈{5,15,25}, and n=750,d=50 indicate a reduction in power for several dependency patterns, whereas DCOR, HSIC, and MEF remained comparatively stable. Nonetheless, UFDM preserved its performance for sparse geometrically structured dependencies (e.g., *Interleaved Moons*), where alternative measures often show more pronounced loss of sensitivity. Due to the high computational cost of UFDM permutation tests, we omitted systematic exploration of these regimes, leaving it to future work. On the other hand, in supervised feature extraction on real datasets, we examined substantially broader (n,d) ranges, including high-dimensional settings such as USPS(n=9298,d=256), Micro-Mass(n=360,d=1300), and Scene(n=2407,d=299). UFDM outperformed one or more baselines on several such datasets (Table 6), suggesting that it may be effective in some larger-dimensional machine learning tasks.
**Future work and potential applications.** Identifying the limit distribution of the empirical UFDM could enable faster alternatives to permutation-based statistical tests, which would also facilitate the systematic analysis of previously mentioned (n,d) settings. However, since the empirical UFDM is not a *U*- or *V*-statistic like HSIC or distance correlation, this would require a non-trivial analysis of the extrema of empirical processes. Possible extensions of UFDM include multivariate generalisations [23] and weighted or normalised variants to enhance empirical stability. From an application perspective, UFDM may prove useful in causality, regularisation, representation learning, and other areas of modern machine learning where statistical dependence serves as an optimisation criterion.

## Figures and Tables

**Table 1:** Empirical power and Wilson CIs for the dependent data (structured dependence patterns) at α=0.05.

Distribution of *Y*	UFDM	DCOR	HSIC	MEF		
Linear (1.0)	1.00 [0.96, 1.00]	1.00 [0.96, 1.00]	1.00 [0.96, 1.00]	1.00 [0.96, 1.00]	d=5	$X∼U{\left[0,1\right]}^{d}$
Linear (0.3)	1.00 [0.96, 1.00]	1.00 [0.96, 1.00]	1.00 [0.96, 1.00]	1.00 [0.96, 1.00]		
Logarithmic	1.00 [0.96, 1.00]	1.00 [0.96, 1.00]	1.00 [0.96, 1.00]	1.00 [0.96, 1.00]		
Quadratic	1.00 [0.96, 1.00]	1.00 [0.96, 1.00]	1.00 [0.96, 1.00]	1.00 [0.96, 1.00]		
Polynomial	1.00 [0.96, 1.00]	1.00 [0.96, 1.00]	1.00 [0.96, 1.00]	1.00 [0.96, 1.00]		
LRSO (0.05)	1.00 [0.96, 1.00]	1.00 [0.96, 1.00]	1.00 [0.96, 1.00]	1.00 [0.96, 1.00]		
Heteroscedastic	1.00 [0.96, 1.00]	1.00 [0.96, 1.00]	1.00 [0.96, 1.00]	1.00 [0.96, 1.00]		
Linear (1.0)	1.00 [0.96, 1.00]	1.00 [0.96, 1.00]	1.00 [0.96, 1.00]	1.00 [0.96, 1.00]	d=15	
Linear (0.3)	1.00 [0.96, 1.00]	1.00 [0.96, 1.00]	1.00 [0.96, 1.00]	1.00 [0.96, 1.00]		
Logarithmic	1.00 [0.96, 1.00]	1.00 [0.96, 1.00]	1.00 [0.96, 1.00]	1.00 [0.96, 1.00]		
Quadratic	1.00 [0.96, 1.00]	1.00 [0.96, 1.00]	1.00 [0.96, 1.00]	1.00 [0.96, 1.00]		
Polynomial	1.00 [0.96, 1.00]	1.00 [0.96, 1.00]	1.00 [0.96, 1.00]	1.00 [0.96, 1.00]		
LRSO (0.05)	1.00 [0.96, 1.00]	1.00 [0.96, 1.00]	1.00 [0.96, 1.00]	1.00 [0.96, 1.00]		
Heteroscedastic	1.00 [0.96, 1.00]	1.00 [0.96, 1.00]	1.00 [0.96, 1.00]	1.00 [0.96, 1.00]		
Linear (1.0)	1.00 [0.96, 1.00]	1.00 [0.96, 1.00]	1.00 [0.96, 1.00]	1.00 [0.96, 1.00]	d=25	
Linear (0.3)	1.00 [0.96, 1.00]	1.00 [0.96, 1.00]	1.00 [0.96, 1.00]	1.00 [0.96, 1.00]		
Logarithmic	1.00 [0.96, 1.00]	1.00 [0.96, 1.00]	1.00 [0.96, 1.00]	1.00 [0.96, 1.00]		
Quadratic	1.00 [0.96, 1.00]	1.00 [0.96, 1.00]	1.00 [0.96, 1.00]	1.00 [0.96, 1.00]		
Polynomial	1.00 [0.96, 1.00]	1.00 [0.96, 1.00]	1.00 [0.96, 1.00]	1.00 [0.96, 1.00]		
LRSO (0.05)	1.00 [0.96, 1.00]	1.00 [0.96, 1.00]	1.00 [0.96, 1.00]	1.00 [0.96, 1.00]		
Heteroscedastic	1.00 [0.96, 1.00]	1.00 [0.96, 1.00]	1.00 [0.96, 1.00]	1.00 [0.96, 1.00]		
Linear (1.0)	1.00 [0.96, 1.00]	1.00 [0.96, 1.00]	1.00 [0.96, 1.00]	1.00 [0.96, 1.00]	d=5	$X∼N(0,I_{d})$
Linear (0.3)	1.00 [0.96, 1.00]	1.00 [0.96, 1.00]	1.00 [0.96, 1.00]	1.00 [0.96, 1.00]		
Logarithmic	1.00 [0.96, 1.00]	1.00 [0.96, 1.00]	1.00 [0.96, 1.00]	1.00 [0.96, 1.00]		
Quadratic	1.00 [0.96, 1.00]	1.00 [0.96, 1.00]	1.00 [0.96, 1.00]	1.00 [0.96, 1.00]		
Polynomial	1.00 [0.96, 1.00]	1.00 [0.96, 1.00]	1.00 [0.96, 1.00]	1.00 [0.96, 1.00]		
LRSO (0.05)	1.00 [0.96, 1.00]	1.00 [0.96, 1.00]	1.00 [0.96, 1.00]	1.00 [0.96, 1.00]		
Heteroscedastic	1.00 [0.96, 1.00]	1.00 [0.96, 1.00]	1.00 [0.96, 1.00]	1.00 [0.96, 1.00]		
Linear (1.0)	1.00 [0.96, 1.00]	1.00 [0.96, 1.00]	1.00 [0.96, 1.00]	1.00 [0.96, 1.00]	d=15	
Linear (0.3)	1.00 [0.96, 1.00]	1.00 [0.96, 1.00]	1.00 [0.96, 1.00]	1.00 [0.96, 1.00]		
Logarithmic	1.00 [0.96, 1.00]	1.00 [0.96, 1.00]	1.00 [0.96, 1.00]	1.00 [0.96, 1.00]		
Quadratic	1.00 [0.96, 1.00]	1.00 [0.96, 1.00]	1.00 [0.96, 1.00]	1.00 [0.96, 1.00]		
Polynomial	1.00 [0.96, 1.00]	1.00 [0.96, 1.00]	1.00 [0.96, 1.00]	1.00 [0.96, 1.00]		
LRSO (0.05)	1.00 [0.96, 1.00]	1.00 [0.96, 1.00]	1.00 [0.96, 1.00]	1.00 [0.96, 1.00]		
Heteroscedastic	1.00 [0.96, 1.00]	1.00 [0.96, 1.00]	1.00 [0.96, 1.00]	1.00 [0.96, 1.00]		
Linear (1.0)	1.00 [0.96, 1.00]	1.00 [0.96, 1.00]	1.00 [0.96, 1.00]	1.00 [0.96, 1.00]	d=25	
Linear (0.3)	1.00 [0.96, 1.00]	1.00 [0.96, 1.00]	1.00 [0.96, 1.00]	1.00 [0.96, 1.00]		
Logarithmic	0.97 [0.92, 0.99]	1.00 [0.96, 1.00]	1.00 [0.96, 1.00]	1.00 [0.96, 1.00]		
Quadratic	1.00 [0.96, 1.00]	1.00 [0.96, 1.00]	1.00 [0.96, 1.00]	1.00 [0.96, 1.00]		
Polynomial	1.00 [0.96, 1.00]	1.00 [0.96, 1.00]	1.00 [0.96, 1.00]	1.00 [0.96, 1.00]		
LRSO (0.05)	1.00 [0.96, 1.00]	1.00 [0.96, 1.00]	1.00 [0.96, 1.00]	1.00 [0.96, 1.00]		
Heteroscedastic	1.00 [0.96, 1.00]	1.00 [0.96, 1.00]	1.00 [0.96, 1.00]	1.00 [0.96, 1.00]		
Linear (1.0)	1.00 [0.96, 1.00]	1.00 [0.96, 1.00]	1.00 [0.96, 1.00]	1.00 [0.96, 1.00]	d=5	X∼Student’st(3)
Linear (0.3)	1.00 [0.96, 1.00]	1.00 [0.96, 1.00]	1.00 [0.96, 1.00]	1.00 [0.96, 1.00]		
Logarithmic	0.98 [0.93, 0.99]	1.00 [0.96, 1.00]	1.00 [0.96, 1.00]	1.00 [0.96, 1.00]		
Quadratic	1.00 [0.96, 1.00]	1.00 [0.96, 1.00]	1.00 [0.96, 1.00]	1.00 [0.96, 1.00]		
Polynomial	0.96 [0.90, 0.98]	1.00 [0.96, 1.00]	1.00 [0.96, 1.00]	1.00 [0.96, 1.00]		
LRSO (0.05)	1.00 [0.96, 1.00]	1.00 [0.96, 1.00]	1.00 [0.96, 1.00]	1.00 [0.96, 1.00]		
Heteroscedastic	1.00 [0.96, 1.00]	1.00 [0.96, 1.00]	1.00 [0.96, 1.00]	1.00 [0.96, 1.00]		
Linear (1.0)	1.00 [0.96, 1.00]	1.00 [0.96, 1.00]	1.00 [0.96, 1.00]	1.00 [0.96, 1.00]	d=15	
Linear (0.3)	1.00 [0.96, 1.00]	1.00 [0.96, 1.00]	1.00 [0.96, 1.00]	1.00 [0.96, 1.00]		
Logarithmic	0.98 [0.93, 0.99]	1.00 [0.96, 1.00]	1.00 [0.96, 1.00]	1.00 [0.96, 1.00]		
Quadratic	0.99 [0.95, 1.00]	1.00 [0.96, 1.00]	1.00 [0.96, 1.00]	1.00 [0.96, 1.00]		
Polynomial	1.00 [0.96, 1.00]	1.00 [0.96, 1.00]	1.00 [0.96, 1.00]	0.99 [0.95, 1.00]		
LRSO (0.05)	1.00 [0.96, 1.00]	1.00 [0.96, 1.00]	1.00 [0.96, 1.00]	1.00 [0.96, 1.00]		
Heteroscedastic	1.00 [0.96, 1.00]	1.00 [0.96, 1.00]	1.00 [0.96, 1.00]	1.00 [0.96, 1.00]		
Linear (1.0)	1.00 [0.96, 1.00]	1.00 [0.96, 1.00]	1.00 [0.96, 1.00]	1.00 [0.96, 1.00]	d=25	
Linear (0.3)	1.00 [0.96, 1.00]	1.00 [0.96, 1.00]	1.00 [0.96, 1.00]	1.00 [0.96, 1.00]		
Logarithmic	0.97 [0.92, 0.99]	1.00 [0.96, 1.00]	1.00 [0.96, 1.00]	1.00 [0.96, 1.00]		
Quadratic	0.98 [0.93, 0.99]	1.00 [0.96, 1.00]	1.00 [0.96, 1.00]	1.00 [0.96, 1.00]		
Polynomial	1.00 [0.96, 1.00]	1.00 [0.96, 1.00]	1.00 [0.96, 1.00]	1.00 [0.96, 1.00]		
LRSO (0.05)	1.00 [0.96, 1.00]	1.00 [0.96, 1.00]	1.00 [0.96, 1.00]	1.00 [0.96, 1.00]		
Heteroscedastic	1.00 [0.96, 1.00]	1.00 [0.96, 1.00]	1.00 [0.96, 1.00]	1.00 [0.96, 1.00]		

**Table 2:** Empirical power with 95% Wilson confidence intervals for dependent data (complex dependence patterns) at α=0.05.

Pattern	UFDM	DCOR	HSIC	MEF	
Mixture Bimodal Marginal	1.00 [0.96, 1.00]	1.00 [0.96, 1.00]	1.00 [0.96, 1.00]	1.00 [0.96, 1.00]	d=5
Mixture Bimodal	1.00 [0.96, 1.00]	1.00 [0.96, 1.00]	1.00 [0.96, 1.00]	1.00 [0.96, 1.00]	
Circular	1.00 [0.96, 1.00]	1.00 [0.96, 1.00]	1.00 [0.96, 1.00]	1.00 [0.96, 1.00]	
Gaussian Copula	1.00 [0.96, 1.00]	1.00 [0.96, 1.00]	1.00 [0.96, 1.00]	1.00 [0.96, 1.00]	
Clayton Copula	1.00 [0.96, 1.00]	1.00 [0.96, 1.00]	1.00 [0.96, 1.00]	1.00 [0.96, 1.00]	
Interleaved Moons	1.00 [0.96, 1.00]	1.00 [0.96, 1.00]	1.00 [0.96, 1.00]	1.00 [0.96, 1.00]	
Mixture Bimodal Marginal	1.00 [0.96, 1.00]	1.00 [0.96, 1.00]	1.00 [0.96, 1.00]	1.00 [0.96, 1.00]	d=15
Mixture Bimodal	1.00 [0.96, 1.00]	1.00 [0.96, 1.00]	1.00 [0.96, 1.00]	1.00 [0.96, 1.00]	
Circular	1.00 [0.96, 1.00]	1.00 [0.96, 1.00]	1.00 [0.96, 1.00]	0.87 [0.79, 0.92]	
Gaussian Copula	1.00 [0.96, 1.00]	1.00 [0.96, 1.00]	1.00 [0.96, 1.00]	1.00 [0.96, 1.00]	
Clayton Copula	1.00 [0.96, 1.00]	1.00 [0.96, 1.00]	1.00 [0.96, 1.00]	1.00 [0.96, 1.00]	
Interleaved Moons	1.00 [0.96, 1.00]	1.00 [0.96, 1.00]	1.00 [0.96, 1.00]	0.49 [0.39, 0.59]	
Mixture Bimodal Marginal	1.00 [0.96, 1.00]	1.00 [0.96, 1.00]	1.00 [0.96, 1.00]	1.00 [0.96, 1.00]	d=25
Mixture Bimodal	1.00 [0.96, 1.00]	1.00 [0.96, 1.00]	1.00 [0.96, 1.00]	1.00 [0.96, 1.00]	
Circular	1.00 [0.96, 1.00]	1.00 [0.96, 1.00]	1.00 [0.96, 1.00]	0.52 [0.42, 0.62]	
Gaussian Copula	0.98 [0.93, 0.99]	1.00 [0.96, 1.00]	1.00 [0.96, 1.00]	1.00 [0.96, 1.00]	
Clayton Copula	1.00 [0.96, 1.00]	1.00 [0.96, 1.00]	1.00 [0.96, 1.00]	1.00 [0.96, 1.00]	
Interleaved Moons	1.00 [0.96, 1.00]	0.97 [0.92, 0.99]	0.96 [0.90, 0.98]	0.27 [0.19, 0.36]	

**Table 3:** UFDM statistic (mean ± std) under true dependence/permuted independence.

Dependence Pattern	d=5	d=15	d=25	
Linear (strong)	0.191±0.035/0.023±0.010	0.208±0.012/0.045±0.009	0.231±0.011/0.083±0.015	$X∼U{\left[0,1\right]}^{d}$
Linear (weak)	0.123±0.035/0.023±0.009	0.143±0.014/0.044±0.011	0.166±0.012/0.081±0.015	
Logarithmic	0.172±0.048/0.023±0.009	0.189±0.022/0.042±0.008	0.195±0.014/0.087±0.015	
Quadratic	0.199±0.048/0.025±0.013	0.200±0.020/0.045±0.009	0.212±0.017/0.082±0.017	
Polynomial	0.185±0.047/0.023±0.010	0.195±0.026/0.047±0.013	0.208±0.015/0.083±0.016	
Contaminated sine	0.059±0.009/0.006±0.002	0.080±0.008/0.009±0.004	0.116±0.007/0.015±0.006	
Conditional variance	0.102±0.024/0.023±0.010	0.142±0.016/0.043±0.010	0.173±0.015/0.080±0.016	
Linear (strong)	0.240±0.013/0.042±0.011	0.239±0.011/0.077±0.014	0.250±0.010/0.101±0.009	$X∼N(0,I_{d})$
Linear (weak)	0.230±0.011/0.044±0.014	0.235±0.013/0.077±0.013	0.244±0.012/0.104±0.013	
Logarithmic	0.254±0.031/0.034±0.010	0.184±0.031/0.051±0.013	0.136±0.023/0.079±0.014	
Quadratic	0.212±0.035/0.028±0.013	0.176±0.025/0.049±0.010	0.146±0.022/0.080±0.014	
Polynomial	0.190±0.041/0.027±0.009	0.176±0.027/0.048±0.011	0.174±0.020/0.073±0.009	
Contaminated sine	0.059±0.009/0.006±0.002	0.082±0.010/0.011±0.005	0.114±0.009/0.014±0.005	
Conditional variance	0.184±0.014/0.038±0.012	0.208±0.013/0.077±0.012	0.218±0.012/0.102±0.013	
Linear (strong)	0.173±0.016/0.031±0.013	0.181±0.017/0.053±0.012	0.207±0.013/0.080±0.013	X∼ Student’s t(3)
Linear (weak)	0.165±0.018/0.030±0.012	0.182±0.018/0.059±0.014	0.205±0.013/0.082±0.012	
Logarithmic	0.150±0.041/0.024±0.011	0.096±0.019/0.041±0.011	0.121±0.026/0.064±0.014	
Quadratic	0.082±0.037/0.014±0.007	0.078±0.020/0.029±0.010	0.097±0.022/0.048±0.012	
Polynomial	0.037±0.022/0.009±0.004	0.050±0.023/0.016±0.008	0.085±0.020/0.033±0.012	
Contaminated sine	0.057±0.008/0.006±0.002	0.078±0.009/0.011±0.004	0.115±0.008/0.014±0.004	
Conditional variance	0.124±0.018/0.027±0.009	0.158±0.014/0.054±0.011	0.180±0.011/0.079±0.013	
Mixture bimodal marginal	0.496±0.007/0.048±0.011	0.500±0.008/0.083±0.012	0.500±0.008/0.101±0.012	Complex patterns
Mixture bimodal	0.883±0.006/0.036±0.013	0.935±0.006/0.047±0.016	0.972±0.005/0.058±0.016	
Circular	0.277±0.023/0.048±0.010	0.259±0.022/0.089±0.012	0.231±0.032/0.114±0.011	
Gaussian copula	0.241±0.011/0.038±0.016	0.248±0.013/0.049±0.013	0.254±0.010/0.056±0.013	
Clayton copula	0.284±0.013/0.038±0.013	0.287±0.013/0.047±0.014	0.290±0.015/0.060±0.014	
Interleaved moons	0.418±0.017/0.020±0.008	0.384±0.024/0.052±0.013	0.339±0.041/0.095±0.014	

**Table 4:** Pairwise wins matrix: entry (i,j) is the number of cases where the method in row *i* outperformed the method in column *j* (Wilcoxon’s signed-rank test, 25 runs, *p*-value threshold 0.05).

	UFDM	DCOR	MEF	HSIC	NCA
UFDM	0	6	4	5	5
DCOR	2	0	3	4	3
MEF	4	8	0	9	7
HSIC	2	4	2	0	3
NCA	5	7	6	8	0

**Table 5:** Classification accuracy comparison. *n* denotes dataset size, $d_{X}$ is input dimensionality, and $n_{c}$ is the number of classes. Best-performing method that is also statistically significant when compared with all other methods (Wilcoxon’s signed-rank test, 25 runs, *p*-value threshold 0.05) is indicated in bold (otherwise, best-performing method is underlined).

Dataset	$\left(n,d_{X},n_{c}\right)$	RAW	UFDM	DCOR	MEF	HSIC	NCA
Australian	(690, 14, 2)	0.710	0.853	0.846	0.850	0.824	0.844
Collins	(500, 22, 2)	0.840	0.926	0.906	0.941	0.927	**0.949**
Heart-statlog	(270, 13, 2)	0.621	0.824	0.823	0.826	0.816	0.817
Mfeat-factors	(2000, 216, 10)	0.783	0.968	0.970	0.968	0.968	0.969
Mfeat-pixel	(2000, 240, 10)	0.946	0.956	0.948	0.957	0.951	**0.959**
Mfeat-zernike	(2000, 47, 10)	0.741	0.812	0.810	0.814	0.811	0.804
Micro-mass	(360, 1300, 10)	0.874	0.925	0.919	0.931	0.923	0.882
Optdigits	(5620, 64, 10)	0.949	0.964	0.961	0.960	0.957	0.963
Parkinsons	(195, 22, 2)	0.756	0.827	0.828	0.850	0.836	0.837
Scene	(2407, 299, 2)	0.886	0.987	0.988	0.953	0.988	0.962
Segment	(2310, 19, 7)	0.760	0.912	0.911	**0.943**	0.936	0.941
Sonar	(208, 60, 2)	0.685	0.745	0.733	0.757	0.734	0.770
Spectf	(349, 44, 2)	0.729	0.737	0.739	0.738	0.739	0.750
USPS	(9298, 256, 10)	0.924	**0.944**	0.941	0.934	0.936	0.940
Wdbc	(569, 30, 2)	0.699	0.948	0.951	0.938	0.900	**0.968**
Wine	(178, 13, 3)	0.552	0.945	0.917	**0.954**	0.947	0.936
WR(b)			20	12	28	11	26
LR(b)			13	25	15	26	18

**Table 6:** Twenty cases (Measures Outperformed) where UFDM outperformed the other baselines.

Dataset	*n*	$d_{X}$	Measures Outperformed
Australian	690	14	DCOR, HSIC, NCA
Collins	500	22	DCOR
Micro-mass	360	1300	NCA
Mfeat-pixel	2000	240	DCOR, HSIC
Mfeat-zernike	2000	47	NCA
Optdigits	5620	64	DCOR, MEF, HSIC
Scene	2407	299	MEF, NCA
USPS	9298	256	DCOR, MEF, HSIC, NCA
Wdbc	569	30	MEF, HSIC
Wine	178	13	DCOR

## Data Availability

All synthetic data were generated as described in the manuscript; real datasets were obtained from OpenML (https://www.openml.org (accessed on 4 December 2025)). Code to reproduce the experiments is available at https://github.com/povidanius/UFDM (accessed on 4 December 2025). No additional unpublished data were used.

## Appendix A

### Appendix A.1. Proofs

In the proofs, we interchangeably abbreviate $\phi_{X}\left(\alpha \right)$ with ϕ(α), $\phi_{Y}\left(\beta \right)$ with ϕ(β), and $\phi_{X,Y}\left(\alpha ,\beta \right)$ with ϕ(γ), where $\gamma ={\left(\alpha^{T},\beta^{T}\right)}^{T}$.

**Proof of Theorem** **1.** Property 1. By Cauchy–Schwarz inequality.

(A1) $$\begin{array}{c} {\left|\Delta \left(\alpha ,\beta \right)\right|}^{2}={\left|E_{X,Y}\left(e^{i\alpha^{T}X}-\phi_{X}\left(\alpha \right)\right)\left(e^{i\beta^{T}Y}-\phi_{Y}\left(\beta \right)\right)\right|}^{2}\leq \\ \leq E_{X}|\left(e^{i\alpha^{T}X}-\phi_{X}\left(\alpha \right)\right){|}^{2}E_{Y}{\left|\left(e^{i\beta^{T}Y}-\phi_{Y}\left(\beta \right)\right)\right|}^{2}. \end{array}$$

Recall that for complex numbers *z* and $z^{\prime }$ we have $|z-z^{\prime }{|}^{2}={\left|z\right|}^{2}-z\;\bar{z^{\prime }}-\bar{z}\;z^{\prime }+{\left|z^{\prime }\right|}^{2}$, where $\bar{z}$ is complex conjugate of *z*. Therefore by plugging $z=e^{i\alpha^{T}X}$ and $z^{\prime }=\phi_{X}\left(\alpha \right)$ from the definition of CF we obtain

$$\begin{array}{c} E_{X}|e^{i\alpha^{T}X}-\phi_{X}{\left(\alpha \right)|}^{2}=1-\phi_{X}\left(\alpha \right)\bar{\phi_{X}\left(\alpha \right)}-\bar{\phi_{X}\left(\alpha \right)}\phi_{X}\left(\alpha \right)+|\phi_{X}{\left(\alpha \right)|}^{2}=1-{\left|\phi_{X}\left(\alpha \right)\right|}^{2}, \end{array}$$

and similarly $E_{Y}|\left(e^{i\beta^{T}Y}-\phi_{Y}\left(\beta \right)\right){|}^{2}=1-{\left|\phi_{Y}\left(\beta \right)\right|}^{2}$. Since the absolute value of CF is bounded by 1, we have that  Equation (A1) is also bounded by 1. Property 2.

$$\begin{array}{c} \mathrm{UFDM}\left(X,Y\right)=\underset{\alpha ,\beta }{\sup}\left|E_{X,Y}e^{i(\alpha^{T}X+\beta_{T}Y)}-E_{X}e^{i\alpha^{T}X}E_{Y}e^{i\beta^{T}Y}\right|\\ =\underset{\beta ,\alpha }{\sup}\left|E_{Y,X}e^{i(\beta^{T}Y+\alpha^{T}X)}-E_{Y}e^{i\beta^{T}Y}E_{X}e^{i\alpha^{T}X}\right|=\mathrm{UFDM}\left(Y,X\right). \end{array}$$

Property 3. Let us assume that X⊥Y. Then $\phi_{X,Y}\left(\alpha ,\beta \right)=E_{X,Y}e^{i(\alpha^{T}X+\beta^{T}Y)}=E_{X}E_{Y}e^{i(\alpha^{T}X+\beta^{T}Y)}=\phi_{X}\left(\alpha \right)\phi_{Y}\left(\beta \right)$. Therefore, UFDM(X,Y)=0. On the other hand, if UFDM(X,Y)=0 then $\phi_{X,Y}\left(\alpha ,\beta \right)=\phi_{X}\left(\alpha \right)\phi_{Y}\left(\beta \right)$ for all $\alpha \in R^{d_{X}}$,$\beta \in R^{d_{Y}}$. Let $\tilde{X}$ and $\tilde{Y}$ be two independent random vectors, having the same distributions as *X* and *Y*, respectively. Therefore $\phi_{X,Y}\left(\alpha ,\beta \right)=\phi_{X}\left(\alpha \right)\phi_{Y}\left(\beta \right)=\phi_{\tilde{X}}\left(\alpha \right)\phi_{\tilde{Y}}\left(\beta \right)=\phi_{\tilde{X},\tilde{Y}}\left(\alpha ,\beta \right)$. The uniqueness of CF [16] implies that distributions of (X,Y) and $\left(\tilde{X},\tilde{Y}\right)$ coincide, from what directly follows that X⊥Y. Property 4. Let $\Sigma_{X,Y}$ be cross-covariance matrix of *X* and *Y*. Since *X* and *Y* are Gaussian, we have $\phi_{X}\left(\alpha \right)=e^{-\frac{1}{2}\alpha^{T}\Sigma_{X}\alpha }$, $\phi_{Y}\left(\beta \right)=e^{-\frac{1}{2}\beta^{T}\Sigma_{Y}\beta }$, $\phi_{X,Y}\left(\alpha ,\beta \right)=e^{-\frac{1}{2}\left(\alpha^{T}\Sigma_{X}\alpha +\beta^{T}\Sigma_{Y}\beta +2\alpha^{T}\Sigma_{X,Y}\beta \right)}$. Therefore, by Equation (11)

(A2) $$\mathrm{UFDM}\left(X,Y\right)=\underset{\alpha ,\beta }{\sup}e^{-\frac{1}{2}\left(\alpha^{T}\Sigma_{X}\alpha +\beta^{T}\Sigma_{Y}\beta \right)}\left|e^{-\alpha^{T}\Sigma_{X,Y}\beta }-1\right|.$$

Property 5. Since $\phi_{AX+a,BY+b}\left(\alpha ,\beta \right)=e^{i\alpha^{T}a+i\beta^{T}b}\phi_{X,Y}\left(A^{T}\alpha ,B^{T}\beta \right)$, and $\phi_{AX+a}\left(\alpha \right)=e^{i\alpha^{T}a}\phi_{X}\left(A^{T}\alpha \right)$, $\phi_{BY+b}\left(\beta \right)=e^{i\beta^{T}b}\phi_{Y}\left(B^{T}\beta \right)$, we have

$$\begin{array}{c} \mathrm{UFDM}\left(AX+a,BY+b\right)=\underset{\alpha ,\beta }{\sup}\left|\phi_{AX+a,BY+b}\left(\alpha ,\beta \right)-\phi_{Ax+a}\left(\alpha \right)\phi_{BY+b}\left(\beta \right)\right|=\\ =\underset{\alpha ,\beta }{\sup}|e^{i\alpha^{T}a+i\beta^{T}b}||\Delta \left(A^{T}\alpha ,B^{T}\beta \right)|=\underset{\alpha ,\beta }{\sup}\left|\Delta \left(A^{T}\alpha ,B^{T}\beta \right)\right|. \end{array}$$

Since both *A* and *B* are full-rank matrices, and $A\in R^{d_{X}\times d_{X}}$, $B\in R^{d_{Y}\times d_{Y}}$, the maximization of the last equation is equivalent to the maximization of |Δ(α,β)|, which by definition is UFDM(X,Y). Property 6. If $A^{\prime }\in R^{d_{X^{\prime }}\times d_{X}}$, $B^{\prime }\in R^{d_{Y^{\prime }}\times d_{Y}}$, $a^{\prime }\in R^{d_{X^{\prime }}}$, $b^{\prime }\in R^{d_{Y^{\prime }}}$ are parameters of linear dimension reduction, where $d_{X^{\prime }}<d_{X}$, and $d_{Y^{\prime }}<d_{Y}$, we have

(A3) $$\mathrm{UFDM}\left(A^{\prime }X+a^{\prime },B^{\prime }Y+b^{\prime }\right)\leq \mathrm{UFDM}\left(AX+a,BY+b\right),$$

for any A,B,a,b of the same dimensions (defined as in Property 5), because maximisation of LHS is conducted in smaller space than that of RHS. By Property 5, it follows that UFDM(AX+a,BY+b)=UFDM(X,Y). Property 7. The independence of *X* and E implies that

$$\begin{array}{c} \mathrm{UFDM}\left(X,f\left(X\right)+\lambda E\right)=\underset{\alpha ,\beta }{\sup}\left|Ee^{i(\alpha^{T}X+\beta^{T}f\left(X\right))}\phi_{E}\left(\lambda \beta \right)-\phi_{X}\left(\alpha \right)\phi_{f(X)}\left(\beta \right)\phi_{E}\left(\lambda \beta \right)\right|, \end{array}$$

which converges to 0, since by multivariate Riemann–Lebesgue lemma [39] the common term $|\phi_{E}\left(\lambda \beta \right)|\to 0$, when λ→∞. The multivariate Riemann–Lebesgue lemma can be applied since E has a density. Property 8. Recall that the total variation distance between joint probability measure $P_{X,Y}$ and product measure $P_{X}P_{Y}$ is given by

$$\mathrm{TV}\left(P_{X,Y},P_{X}P_{Y}\right)=\frac{1}{2}\int \left|p_{X,Y}\left(x,y\right)-p_{X}\left(x\right)p_{Y}\left(y\right)\right|dxdy,$$

where $p_{X,Y}\left(x,y\right)$ is joint density, and $p_{X}\left(x\right)$, $p_{Y}\left(y\right)$ are marginal ones. Recall that Pinsker’s inequality for total variation states that $\mathrm{TV}\left(P_{X,Y},P_{X}P_{Y}\right)\leq \sqrt{\frac{1}{2}\mathrm{MI}\left(X,Y\right)}$, where MI(X,Y) is mutual information between *X* and *Y*. Therefore,

$$\begin{array}{c} \left|\Delta (\alpha ,\beta )\right|=\left|\int e^{i(\alpha^{T}x+\beta^{T}y)}\left(p_{X,Y}\left(x,y\right)-p_{X}\left(x\right)p_{Y}\left(y\right)\right)dxdy\right|\\ \leq \int \left|p_{X,Y}\left(x,y\right)-p_{X}\left(x\right)p_{Y}\left(y\right)\right|dxdy=2\mathrm{TV}\left(P_{X,Y},P_{X}P_{Y}\right). \end{array}$$

By taking the supremum we have $\mathrm{UFDM}\left(X,Y\right)\leq \min\left\{1,2\mathrm{TV}\left(P_{X,Y},P_{X}P_{Y}\right)\right\}\leq \min\left\{1,\sqrt{2\mathrm{MI}(X,Y)}\right\}$ by Property 1 and Pinsker’s inequality. Property 9. Independence condition $Z⊥{\left(X^{⊤},Y^{⊤}\right)}^{⊤}$ gives

$$\Delta_{{\left(X^{⊤},Z^{⊤}\right)}^{⊤},Y}\left(\alpha_{X},\alpha_{Z},\beta \right)=\varphi_{Z}\left(\alpha_{Z}\right)\;\Delta_{X,Y}\left(\alpha_{X},\beta \right).$$

Since $|\varphi_{Z}\left(\alpha_{Z}\right)|\leq 1$ and $|\varphi_{Z}\left(0\right)|=1$, we have $\sup_{\alpha_{X},\alpha_{Z},\beta }\left|\Delta_{{\left(X^{⊤},Z^{⊤}\right)}^{⊤},Y}\right|=\sup_{\alpha_{X},\beta }\left|\Delta_{X,Y}\left(\alpha_{X},\beta \right)\right|.$ Therefore, $\mathrm{UFDM}\left({\left(X^{⊤},Z^{⊤}\right)}^{⊤},Y\right)=\mathrm{UFDM}\left(X,Y\right)$.    □

**Proof of Proposition** **2.** Let ϵ>0. Since ECF is CF, and a product of two CFs also is CF, by Theorem 2 and triangle inequality, we can find natural number $n_{0}$ such that $\forall n>n_{0}$: $||\Delta -\Delta_{n}{\left|\right|}_{L_{\infty }}^{t_{n}}=\sup_{||\gamma ||<t_{n}}|\Delta \left(\gamma \right)-\Delta_{n}\left(\gamma \right)|=\sup_{||\gamma ||<t_{n}}|\phi \left(\gamma \right)-\psi \left(\gamma \right)-\phi_{n}\left(\gamma \right)+\psi_{n}\left(\gamma \right)|=\sup_{||\gamma ||<t_{n}}|\phi \left(\gamma \right)-\phi_{n}\left(\gamma \right)+\psi_{n}\left(\gamma \right)-\psi \left(\gamma \right)|\leq \sup_{||\gamma ||<t_{n}}|\phi \left(\gamma \right)-\phi_{n}\left(\gamma \right)|+\sup_{||\gamma ||<t_{n}}\left|\psi \left(\gamma \right)-\psi_{n}\left(\gamma \right)\right|\leq \epsilon$, almost surely. From the inverse triangle inequality for norms we have ${\left|||\Delta |\right|}_{L_{\infty }}^{t_{n}}-\left|\right|\Delta_{n}{\left|\right|}_{L_{\infty }}^{t_{n}}|\leq ||\Delta -\Delta_{n}{\left|\right|}_{L_{\infty }}^{t_{n}}\leq \epsilon$, almost surely. On the other hand, along with the definition of $\mathrm{UFDM}\left(X,Y\right)=\lim_{n\to \infty }{\left||\Delta \left(\gamma \right)|\right|}_{L_{\infty }}^{t_{n}}$, this implies that $\left|\mathrm{UFDM}\left(X,Y\right)-|\right|\Delta_{n}{\left|\right|}_{L_{\infty }}^{t_{n}}{\left|\leq |\mathrm{UFDM}\left(X,Y\right)-||\Delta |\right|}_{L_{\infty }}^{t_{n}}{\left|+|||\Delta |\right|}_{L_{\infty }}^{t_{n}}-\left|\right|\Delta_{n}{\left|\right|}_{L_{\infty }}^{t_{n}}|$ will be arbitrarily small almost surely, when *n* is sufficiently large.    □

### Appendix A.2. Proof of Theorem 3

**Proof of Theorem** **3.** Recall that $Z={\left(X^{T},Y^{T}\right)}^{T}$, $\gamma ={\left(\alpha^{T},\beta^{T}\right)}^{T}\in R^{d}$ with $d=d_{X}+d_{Y}$ and

$$\Delta \left(\gamma \right)\;=\;\phi \left(\gamma \right)-\psi \left(\gamma \right),\;\Delta_{n}\left(\gamma \right)\;=\;\phi_{n}\left(\gamma \right)-\psi_{n}\left(\gamma \right).$$

**Step 1. Lipschitz continuity.** First, we will prove that Δ(γ) and $\Delta_{n}\left(\gamma \right)$ are Lipschitz continuous. For the population version, consider

$$|\Delta \left(\gamma \right)-\Delta \left(\gamma^{\prime }\right)|\leq |\phi \left(\gamma \right)-\phi \left(\gamma^{\prime }\right)|+|\psi \left(\gamma \right)-\psi \left(\gamma^{\prime }\right)|.$$

Since $\phi \left(\gamma \right)=E\exp\left(i\gamma^{T}Z\right)$, by inequality $|e^{ia}-e^{ib}\left|\leq |a-b\right|$, a,b∈R

$$|\phi \left(\gamma \right)-\phi \left(\gamma^{\prime }\right)|\leq E|\exp\left(i\gamma^{T}Z\right)-\exp\left(i\gamma^{\prime T}Z\right)\left|\leq E\right|{\left(\gamma -\gamma^{\prime }\right)}^{T}Z|\leq ∥\gamma -\gamma^{\prime }∥E∥Z∥.$$

Similarly,

$$\begin{array}{cc} \left|\psi \left(\gamma \right)-\psi \left(\gamma^{\prime }\right)\right|=\left|\phi \left(\alpha \right)\phi \left(\beta \right)-\phi \left(\alpha^{\prime }\right)\phi \left(\beta^{\prime }\right)\right|=\left|\phi (\alpha )\right|\left|\phi \left(\beta \right)-\phi \left(\beta^{\prime }\right)\right|+\left|\phi (\beta^{\prime })\right|\left|(\phi \left(\alpha \right)-\phi \left(\alpha^{\prime }\right)\right| & \\ \leq |\phi \left(\alpha \right)-\phi \left(\alpha^{\prime }\right)|+|\phi \left(\beta \right)-\phi \left(\beta^{\prime }\right)|, \end{array}$$

since |ϕ(α)|≤1, |ϕ(β)|≤1. Therefore,

$$|\phi \left(\alpha \right)-\phi \left(\alpha^{\prime }\right)|\leq E∥X∥∥\alpha -\alpha^{\prime }∥,\;|\phi \left(\beta \right)-\phi \left(\beta^{\prime }\right)|\leq E∥Y∥∥\beta -\beta^{\prime }∥.$$

Thus,

$$|\psi \left(\gamma \right)-\psi \left(\gamma^{\prime }\right)|\leq (E∥X∥+E∥Y∥)∥\gamma -\gamma^{\prime }∥,$$

so Δ(γ) is Lipschitz with constant L=E∥Z∥+E∥X∥+E∥Y∥<∞. For the empirical version,

$$|\Delta_{n}\left(\gamma \right)-\Delta_{n}\left(\gamma^{\prime }\right)\left|\leq \right|\phi_{n}\left(\gamma \right)-\phi_{n}\left(\gamma^{\prime }\right)\left|+\right|\psi_{n}\left(\gamma \right)-\psi_{n}\left(\gamma^{\prime }\right)|,$$

where

$$|\phi_{n}\left(\gamma \right)-\phi_{n}\left(\gamma^{\prime }\right)|\leq \frac{1}{n}\sum_{j=1}^{n}|\gamma^{T}Z_{j}-\gamma^{\prime T}Z_{j}|\leq \left(\frac{1}{n}\sum_{j=1}^{n}∥Z_{j}∥\right)∥\gamma -\gamma^{\prime }∥,$$

and

$$|\psi_{n}\left(\gamma \right)-\psi_{n}\left(\gamma^{\prime }\right)\left|\leq \right|\phi_{n}\left(\alpha \right)-\phi_{n}\left(\alpha^{\prime }\right)\left|+\right|\phi_{n}\left(\beta \right)-\phi_{n}\left(\beta^{\prime }\right)|\leq \frac{1}{n}\left(\sum_{j=1}^{n}∥X_{j}∥+\sum_{j=1}^{n}∥Y_{j}∥\right)∥\gamma -\gamma^{\prime }∥.$$

Define $L_{n}=\frac{1}{n}\sum_{j=1}^{n}(∥Z_{j}∥+∥X_{j}∥+∥Y_{j}∥)$, so $\Delta_{n}\left(\gamma \right)$ is Lipschitz with random constant $L_{n}$. Recall that $EL_{n}=L$, $E{\left(L_{n}-L\right)}^{2}=\sigma^{2}/n$ are finite because of bounded second moment assumption. $L_{n}$ concentrates around *L*, and by Cantelli’s inequality, we have

(A4) $$\mathrm{Pr}\left(L_{n}\geq 2L\right)=\mathrm{Pr}\left(L_{n}-L\geq L\right)\leq \frac{1}{1+n{\left(L/\sigma \right)}^{2}}\leq \frac{\sigma^{2}}{nL^{2}}.$$

**Step 2. Construct a δ-net and bound the deviation on the δ-net.** For $B_{t}=\left\{\gamma :∥\gamma ∥<t\right\}$, construct a δ-net $\left\{\gamma_{1},\ldots ,\gamma_{N(t,\delta )}\right\}$ such that every $\gamma \in B_{t}$ is within δ of some $\gamma_{k}$. The cardinality satisfies $N\left(t,\delta \right)\leq {\left(3t/\delta \right)}^{d}$ [40]. For fixed $\gamma_{k}$, bound $|\Delta_{n}\left(\gamma_{k}\right)-\Delta \left(\gamma_{k}\right)|$. Changing one $Z_{j}$ to $Z_{j}^{\prime }$ alters $\phi_{n}\left(\gamma_{k}\right)$ by at most 2/n, $\phi_{n}\left(\alpha_{k}\right)$ and $\phi_{n}\left(\beta_{k}\right)$ by at most 2/n each, and $\psi_{n}\left(\gamma_{k}\right)$ by at most 4/n. Thus, $|\Delta_{n}\left(\gamma_{k}\right)-\Delta_{n}^{\prime }\left(\gamma_{k}\right)|\leq 6/n$. By McDiarmid’s inequality,

$$\mathrm{Pr}(|\Delta_{n}\left(\gamma_{k}\right)-E\Delta_{n}\left(\gamma_{k}\right)\left|>u\right)\leq 2\exp\left(-\frac{nu^{2}}{18}\right).$$

Compute the bias: $E\phi_{n}\left(\gamma_{k}\right)=\phi \left(\gamma_{k}\right)$, and $E\psi_{n}\left(\gamma_{k}\right)=\frac{1}{n}\phi \left(\gamma_{k}\right)+\left(1-\frac{1}{n}\right)\psi \left(\gamma_{k}\right),$ so

$$E\Delta_{n}\left(\gamma_{k}\right)=\left(1-\frac{1}{n}\right)\Delta \left(\gamma_{k}\right),\;\left|E\Delta_{n}\left(\gamma_{k}\right)-\Delta \left(\gamma_{k}\right)\right|\leq \frac{1}{n}.$$

Thus,

$$\mathrm{Pr}(|\Delta_{n}\left(\gamma_{k}\right)-\Delta \left(\gamma_{k}\right)\left|>\varepsilon \right)\leq 2\exp\left(-\frac{n}{18}{\left(\varepsilon -\frac{1}{n}\right)}^{2}\right),\;\varepsilon >\frac{1}{n}.$$

**Step 3. Extend to the entire frequency ball.** For any $\gamma \in B_{t}$, choose $\gamma_{k}$ with $∥\gamma -\gamma_{k}∥\leq \delta$. Then we have

(A5) $$\begin{array}{c} |\Delta_{n}\left(\gamma \right)-\Delta \left(\gamma \right)|\leq |\Delta_{n}\left(\gamma \right)-\Delta_{n}\left(\gamma_{k}\right)\left|+\right|\Delta_{n}\left(\gamma_{k}\right)-\Delta \left(\gamma_{k}\right)|+|\Delta \left(\gamma_{k}\right)-\Delta \left(\gamma \right)| \end{array}$$

(A6) $$\begin{array}{c} \leq L_{n}\delta +\left|\Delta_{n}\left(\gamma_{k}\right)-\Delta \left(\gamma_{k}\right)\right|+L\delta . \end{array}$$

Thus, $\sup_{\gamma \in B_{t}}|\Delta_{n}\left(\gamma \right)-\Delta \left(\gamma \right)|\leq \left(L_{n}+L\right)\delta +\max_{k}\left|\Delta_{n}\left(\gamma_{k}\right)-\Delta \left(\gamma_{k}\right)\right|$. Then by union bound

(A7) $$\begin{array}{cc} \mathrm{Pr}\left(\underset{\gamma \in B_{t}}{\sup}\left|\Delta_{n}\left(\gamma \right)-\Delta \left(\gamma \right)\right|>\varepsilon \right)\; & \leq \mathrm{Pr}\left(\left(L_{n}+L\right)\delta >\frac{\varepsilon }{2}\right)\\ \; & \;+\mathrm{Pr}\left(\max_{k}\left|\Delta_{n}\left(\gamma_{k}\right)-\Delta \left(\gamma_{k}\right)\right|>\frac{\varepsilon }{2}\right). \end{array}$$

Recall that in Equation (A4) we showed that $\mathrm{Pr}\left(L_{n}>2L\right)\leq \frac{\sigma^{2}}{nL^{2}}$. Choosing $\delta =\frac{\varepsilon }{6L}$ implies

(A8) $$\mathrm{Pr}\left(\left(L_{n}+L\right)\delta >\frac{\varepsilon }{2}\right)=\mathrm{Pr}\left(L_{n}>2L\right)\leq \frac{\sigma^{2}}{nL^{2}}.$$

For the max term, by the union bound,

(A9) $$\mathrm{Pr}(\max_{k}|\Delta_{n}\left(\gamma_{k}\right)-\Delta \left(\gamma_{k}\right)\left|>\frac{\varepsilon }{2}\right)\leq 2N\left(t,\delta \right)\exp\left(-\frac{n}{18}{\left(\frac{\varepsilon }{2}-\frac{1}{n}\right)}^{2}\right),$$

where $N\left(t,\delta \right)\leq {\left(3t/\delta \right)}^{d}={\left(\frac{18tL}{\varepsilon }\right)}^{d}$.

**Step 4: Final bound.** Plugging Equation (A8) and Equation (A9) into Equation (A7) we have

$$\mathrm{Pr}(\underset{\gamma \in B_{t}}{\sup}|\Delta_{n}\left(\gamma \right)-\Delta \left(\gamma \right)\left|>\varepsilon \right)\leq 2{\left(\frac{Ct}{\varepsilon }\right)}^{d}\exp\left(-\frac{n}{18}{\left(\frac{\varepsilon }{2}-\frac{1}{n}\right)}^{2}\right)+\frac{\sigma^{2}}{nL^{2}}.$$

Finally, the stated bound follows from the inverse triangle inequality for norms.   □

### Appendix A.3. Ablation Experiment on SVD Warm-Up

**Table A1:** *p*-value means and standard deviations of the analysed dependence patterns in permutation tests for UFDM without SVD warm-up (Algorithm 2), uniformly initialising parameters α and β from [−1,1] interval. Here $X∼N(0,I_{d})$.

Distribution of *Y*	d=5	d=15	d=25
Linear (1.0)	0.002 ± 0.000	0.002 ± 0.000	0.002 ± 0.000
Linear (0.3)	0.002 ± 0.000	0.002 ± 0.000	0.002 ± 0.000
Logarithmic	0.035 ± 0.097	0.192 ± 0.251	0.387 ± 0.297
Quadratic	0.023 ± 0.061	0.298 ± 0.291	0.285 ± 0.145
Polynomial	0.002 ± 0.000	0.062 ± 0.134	0.056 ± 0.078
LRSO (0.05)	0.002 ± 0.001	0.041 ± 0.066	0.026 ± 0.040
Heteroscedastic	0.004 ± 0.006	0.002 ± 0.001	0.003∗ ± 0.003

### Appendix A.4. Dependency Patterns

**Table A2:** Dependence structures. Lin[a,b] denotes uniform linear spacing over given interval [a,b], a<b, X⊥E∼N(0,I), and *d* is dimension. Fixed parameters k=6, ρ=0.85, θ=5.0. By ⊙ we denote element-wise product.

Type	Formula
Structured dependence patterns ($X∼\{N\left(0,I_{d}\right),U{\left[0,1\right]}^{d},\mathrm{Student}\;t_{3}\left(0,I_{d}\right)\}$)	
Linear(p)	Y=pWX+0.1E, p∈R
Logarithmic	Y=log(1.0+WX⊙WX)+0.1E
Quadratic	Y=WX⊙WX+0.1E
Cubic	Y=0.5(WX⊙WX⊙WX)−WX⊙WX+0.1E
LRSO(p)	$\begin{array}{c} X_{0}∼P_{X},\;Y_{0}=\sin\left(k\left(w^{T}X_{0}\right)\right)1_{d}+0.1E\;(\mathrm{proportion}\;1\;-\;p)\\ X_{1}⊥Y_{1}∼N\left(0,\;25^{2}I_{d}\right)\;(\mathrm{proportion}\;p),\\ \left(X,Y\right)=\mathrm{random}\text{-}\mathrm{shuffle}\left(X_{0}\cup X_{1},\;Y_{0}\cup Y_{1}\right) \end{array}$
Heteroscedastic	$Y=(1.0+E_{1})WX+0.1E$, $E_{1}∼N\left(0,I\right)$
Complex dependence patterns	
Bimodal	$\begin{array}{c} S∼\mathrm{Uniform}(\{-1,1\})\\ \mu_{X}=21_{d_{X}},\;\mu_{Y}=21_{d_{Y}}\\ X∼N(S\mu_{X},I_{d_{X}})\\ Y∼N(S\mu_{Y},I_{d_{Y}}) \end{array}$
Sparse bimodal	$X∼0.5\;N\left(\mu ,I_{d}\right)+0.5\;N\left(-\mu ,I_{d}\right)$, μ=(2,0,…,0)
Sparse circular	$\begin{array}{c} T∼Lin\left[0,2\pi \right],\;R∼N\left(1,0.2^{2}\right)\\ X=\left(R\cos T,R\sin T,\eta \right),\;\eta ∼N\left(0,I_{d-2}\right)\\ Y=\left(R\cos\left(T+\delta \right),R\sin\left(T+\delta \right),\zeta \right)+0.1E,\;\delta ∼N\left(0,1\right),\;\zeta ∼N\left(0,I_{d-2}\right) \end{array}$
Gaussian copula	Marginals $∼N(0,\rho 1_{d\times d}+\left(1-\rho \right)I_{d})$.
Clayton copula	Parameterθandstandardnormalmarginalsforeachcomponent.
Interleaved Moons	$\begin{array}{c} \left(X_{0},L_{X}\right)=\mathrm{make}\text{\_}\mathrm{moos}\left(\right),\;\left(Y_{0},L_{Y}\right)=\mathrm{make}\text{\_}\mathrm{moos}\left(\right)\\ \mathrm{For}\;\mathrm{each}\;\mathrm{sample}\;i:\\ \;X_{i}^{\left(1,2\right)}={\left(X_{0}\right)}_{i}\\ \;Y_{i}^{\left(1,2\right)}∼\mathrm{Uniform}\left\{{\left(Y_{0}\right)}_{j}|{\left(L_{Y}\right)}_{j}\neq {\left(L_{X}\right)}_{i}\right\}\\ \;X_{i}^{\left(3:d\right)},Y_{i}^{\left(3:d\right)}∼N\left(0,I_{d-2}\right) \end{array}$

We used sklearn.datasets.make_moons.
